# Associations of dietary methyl donor nutrients with common psychological conditions (depression, anxiety and stress) among reproductive-aged women in Kabul, Afghanistan

**DOI:** 10.1186/s40795-023-00796-9

**Published:** 2023-11-23

**Authors:** Fawzia Zahidi, Hanieh Abbasi, Pamela J Surkan, Leila Azadbakht

**Affiliations:** 1 Department of Community Nutrition, School of Nutritional Sciences and Dietetics, Tehran University of Medical Sciences, P. O. Box: 1416643931, Tehran, Iran; 2 Department of International Health, John Hopkins School of Public Health, Baltimore, MD USA; 3 Diabetes Research Center, Endocrinology and Metabolism Clinical Sciences Institute, Tehran University of Medical Sciences, Tehran, Iran

**Keywords:** Women, Methyl donor nutrients, Depression, Anxiety, Distress

## Abstract

**Background:**

Higher levels of methyl donor nutrients may be associated with better psychological conditions. Little is known about the association of methyl donor nutrients with psychological conditions among women especially in Asian countries such as Afghanistan.

**Method:**

This cross-sectional study was conducted in Kabul, Afghanistan to assess the association of methyl donor nutrients with common psychological conditions (depression, anxiety and stress) among reproductive-aged women using multistage random sampling to choose one health center from each municipality out of four cardinal directions. Finally a sample of 421 reproductive-aged women with a mean BMI of 23.3 ± 5.0 kg/m2 and an age range of 15–45 years were collected. All women’s dietary intakes were obtained using a 24-recall questionnaire. Depression, Anxiety and Stress Scale − 21 Items (DASS-21) was used to assess psychological conditions. Chi-square tests and one-way ANOVAs were performed to assess general characteristics. Residual model test while adjusting for energy intake was used to assess nutrient intake of methyl donor nutrients and food groups. We fitted logistic regression models to assess risk for Common mental health problems (CMHPs) based on methyl donor tertiles.

**Result:**

We observed that there is no significant association between methyl donor nutrients and psychological disorders in both crude and adjusted models (depression, OR = 0.95, CI: 0.48; 1.88; anxiety, OR = 0.88, CI: 0.43, 1.79; stress, OR = 0.73, CI: 0.38, 1.40), (*p* > 0.05).

**Conclusion:**

Overall, we did not find any significant association between methyl donor nutrients and depression, anxiety and stress.

## Introduction

Common mental health problems (CMHPs) include psychological stress, anxiety and depression .The global prevalence of depression has been estimated to be 5.0% in adults, 5.7% in the elderly and 3.8% in the total population [1]. Consistently, the prevalence of depression is higher among women than men [1, 2]. In 2015, the World Health Organization (WHO) reported that worldwide nearly 264 million people were suffering from anxiety [1]. According to a meta-analysis conducted in 2019, the global prevalence of distress was 50% in the general population [3].

In a population-based study of mental health and disability among adults in Afghanistan, the prevalence of depression, anxiety, and posttraumatic stress disorder (PTSD), was 68%, 72%, and 42%, respectively. These mental health problems were more common in women than in men [4]. Mental disorders can lead to other complicated health conditions such as hypertension, heart attacks, obesity and addiction [2, 5].

While many individual factors are associated with mental disorders, dietary patterns may also have a critical role in the incidence and prevention of depression and anxiety [6]. Previous studies have reported that dietary factors such as w3-fatty acids [6, 7], proteins, minerals (e.g. zinc, iron, iodine) [7] and methyl donor nutrients (vitamins B2, B6, folate, B12, methionine and betaine) [7, 8] are protective against mental disorders and improve brain functioning.

Studies have shown that methyl donor nutrients play either direct or indirect roles in physiological mechanisms that lead to synaptic plasticity, cholinergic signalling, membrane integrity, DNA and histone methylation, thereby improve brain structure and function [8, 9]. Bjelland et al., determined that high intake of vitamin B12 and folate were associated with lower incidence of depression in Norwegian adults [10]. A meta-analysis on the association of B2, B6 and B12 with the risk of depression found significant inverse associations between dietary intakes of these vitamins and depression in women [11]. Kafeshani et al., in a study of vitamin B6 intake in relation to depression and anxiety among adults in Iran, determined that lower intake of vitamin B6 was associated with increased risk of depression and anxiety among women [12].

To date, while some studies on the association of dietary methyl donor nutrients and common mental disorders exist, they have mostly been conducted in developed countries. In Afghanistan as a post-conflict country there is still a scarcity of data on relation to methyl donor nutrients and mental health problems. Therefore, we aimed to determine how methyl donor nutrients are associated with mental health problems among reproductive-aged women in Kabul, Afghanistan. Moreover, we aimed to find the association between methyl donor intake and intakes of other food macronutrients and micronutrients as well as the prevalence of different psychological conditions among aforementioned population.

## Methods

This cross-sectional study was conducted in Kabul, a city with more than 160 health facilities and 22 municipalities [13]. We used a multistage random sampling technique, in each zone of Kabul (North, East, West, and South), we first randomly selected one municipality and from each municipality we randomly selected one health centre. Three comprehensive health centers (CHCs) were selected from the 15th (North), 13th (West), and 9th municipalities (East) [14]. One district hospital was selected from the 16th municipality (south) [14]. From these health centers, we took a convenience sample of 421 reproductive-aged women. Equal samples of women of reproductive age (n = 105) were included from three of the health centres and (n = 106) from one health center according to the following formula [15]:

α = 0.05.

Z = 95% (1.96)

*P* = 47 (*P* = 47%, the prevalence of overweight among wealthy reproductive-aged women) [16].

q= (1-0.47) = (0.53)

$$n\, = \,\frac{{{{({Z_{1 - \frac{\alpha }{2}}})}^2}pq}}{{{{(r)}^2}}}\, = \,\frac{{{{(1.96)}^2}\,0.47\,x\,0.53}}{{{{(0.05)}^2}}}\, = \,382.7\, \approx \,383$$

For the non-response coverage of the study, we add the 10% to our sample size, which is calculated as 10% of participants= (10*383)/100 = 38 people. We then added 38 to our total sample size, 38 + 383 = 421 participants. An equal number of routine attending women (n = 105) were sampled from each of the four health centres. Women of reproductive age (15–45 years) who agreed to take part in this study and who were clients of selected clinics, as well as women with no dietary restrictions, were included in this study. We excluded women of reproductive age who had previously been diagnosed with a mental disorder by a psychiatrist.

### Assessment of dietary intake

All women’s dietary intakes were assessed using a 24-hour recall questionnaire ,which is a reliable and validated method [17, 18]. Dietary intakes were collected on three days of the week, on two weekdays and on one weekend day [19]. To facilitate the data collection, interviewers used a variety of tools to determine serving sizes, including can sizes, a chunk of bread that could fit in the palm of a hand, tablespoons, teaspoons, ladles, plates, bowls, glasses, and photographs of common household meals. Portions sizes were estimated based on household eating/cooking equipment and quantities were reported. Then, the quantities were entered into Nutritionist 4 (NUT4) software for nutrient adequacy analysis and were converted into grams. Total and mean intakes of each food and nutrient consumed were then calculated.

### Assessment of anthropometric indices

Anthropometric indices, such as weight, height, waist circumference and body mass index (BMI) were measured and computed for all participants. BMI was calculated by dividing weight (kg) by height^2^ (m). A calibrated digital scale (SECA 831, Germany) was used for weight measurements. Waist circumference (WC) was measured using an unstretched flexible anthropometric tape at a point midway between the lowest rib and the upper edge of the iliac crest, after normal expiration and without applying pressure to the body surface. We used the World Health Organization (WHO) adult BMI classifications: BMI < 18.5 for low weight, BMI = 18.5 to 24.9 for normal weight, BMI = 25 to 29.9 for overweight, and BMI ≥ 30 for obesity [20].

### Assessment of common mental health problems

We used the Depression, Anxiety and Stress Scale − 21 Items (DASS-21), a set of self-report scales to assess symptoms of depression, anxiety and stress. The DASS-21 scale has one section for depression, another for anxiety and a final section for stress (each section contains seven items) [21]. Depressive symptoms were assessed by evaluating dysphoria (a feeling of general dissatisfaction with life), hopelessness, devaluation of one’s life, self-deprecation, lack of interest, anhedonia (inability to experience pleasure), and inertia (a tendency to do nothing). The anxiety component includes autonomic arousal, skeletal muscular responses, and a subjective feelings of anxious affect. The stress component is sensitive to persistent non-specific stimulant levels. The reliability and validity of this instrument have been measured both locally and internationally and is available in 34 languages [22].

The CMHPs have been categorized into three categories: normal, moderate, and severe. The cut-off point for depression is (normal < 13, moderate = 14 to 21, and severe > 22), for anxiety ((normal < 9, moderate = 10 to 14, and severe > 15), and for stress (normal < 18, moderate = 19 to 25, and severe > 26).

### Assessment of methyl donor nutrients intake

Dietary intake of methyl donor nutrients (Vitamin B2, B6, folate, B12, methionine, betaine and choline) were calculated using Nutritionist 4 (NUT4) software. The aforementioned nutrients were extracted for each participant and then each of these nutrients were calculated for each individual from the 1st up to the 10th decile. Total scores were derived by summing the scores of each participant (range 7 to 68) and categorized into tertiles. We considered the cut-off points Q1 < 23, 46 < Q2 > 23 and > 46 for Q3. We then considered demographic variables such as women’s age, level of education, monthly household income, marital status, place of residence, history of disease (blood pressure and diabetes) among the tertiles.

### Physical activity questionnaire (IPAQ)

The IPAQ is a self-report questionnaire developed in 1998. The reliability and validity of this questionnaire was tested in 12 countries in 2000. The final results suggest that it is an acceptable measure in many countries and in different languages. We used the long form of the IPAQ, which contains four sections (work, cycling/transport, house/garden and leisure) with 27 items [23] and three categories of vigorous, moderate and light physical activity in the last 7 days [24].

### Statistical analysis

The quantity of nutrients consumed by each participant was calculated using Nutritionist IV software. The data were analyzed using the Statistical Package for Social Science (SPSS Version 26) software. Regarding individuals’ general characteristics, Chi-square tests and one-way ANOVAs were performed. To assess nutrient intake of methyl donor nutrients and food groups, we used the residual model test while adjusting for energy intake. We fitted logistic regression models to assess the risk of CMHPs based on methyl donor tertiles.‎.

## Results

We included 421 reproductive-aged women with a mean BMI of 23.3 ± 5.0 kg/m^2^ and an age range of 15–45 years. Approximately 70% of the participants were married. Table 1 shows the general characteristics of the participants according to the tertiles of methyl donor nutrients. Women in the top tertile had significantly higher income $278.1 ± 212.7 compared to women in the lowest tertile $188.2 ± 173.8 (*p* < 0.05). In contrast, women in the third tertile had lower weight (55.8 ± 9.7 kg), waist circumferences (33.0 ± 4.2), BMI (23.0 ± 3.7 kg/m^2^) and age (30.4 ± 8.6 years). In addition, participants in the top tertile were more likely to be married, more educated, and less likely to have diabetes and hypertension.

**Table 1:** General participant characteristics across tertiles of methyl donor nutrients

Tertiles of methyl donor micronutrients										
**Variables**	T1(n = 145)			T2 (n = 137)			T3 (139)			*P*-value^#^
	N (%)	Mean ± SD*		N (%)	Mean ± SD		N (%)	Mean ± SD		
Age (year)		32.1 ± 9.2			30.5 ± 9.2			30.4 ± 8.6		0.26
Income ($)		188.2 ± 173.8			260.5 ± 215.0			278.1 ± 212.7		< 0.001
Weight (Kg)		56.3 ± 9.1			58.4 ± 46.4			55.8 ± 9.7		0.711
BMI (kg/m^2^)		23.5 ± 4.9			23.5 ± 6.2			23.0 ± 3.7		0.651
Waist circumstance (cm)		36 ± 14.6			36.4 ± 21.7			33.0 ± 4.2		0.113
Marital status										
Single Married Widowed	24 (22.4) 112 (38.2) 9 (42.9)			46 (43.0) 84 (28.7) 7 (33.3)			37 (34.6) 97 (33.1) 5 (23.8)			0.020
Education level > high school	31 (1.3)			44 (32.1)			53 (38.1)			0.028
Settlement area										
East West North South	26 (24.8) 79 (75.2) 20 (18.9) 20 (19.0)			39 (37.1) 23 (21.9) 37 (34.9) 38 (36.2)			40 (38.1) 3 (2.9) 49 (46.2) 47 (44.8)			< 0.001
Illness history										
Diabetes	4 (28.6)			4 (28.6)			6 (42.9)			0.726
Hypertension	17 (51.5)			6 (18.2)			10 (30.3)			0.068
Physical activity										
Moderat Intense	118 (34.6) 27 (33.7)			108 (31.7) 29 (36.2)			115 (33.7) 24 (30.0)			0.637

* Number, percentages, mean values and standard deviation, n = 421 are presented. The values mean ± standard deviation (SD) were obtained from one way ANOVAs.

^#^*P*-values were obtained from Chi-Square tests.

Table 2 shows the dietary intake of selected nutrients, methyl donor nutrients and food groups. Participants in top tertiles had significantly higher intakes of energy, fat, protein, carbohydrate, vitamins (B2, B6, B9, B12), methionine, betaine, choline, iron, calcium and zinc. In contrast they had lower intakes of cereals and vegetables compared to participants in the first and second tertiles.

**Table 2:** Intakes of food groups and nutrients of study participants across the tertiles of methyl nutrients

Tertiles of methyl donor micronutrients					
Variables	RDA^9*^	T1(n = 145)	T2 (n = 137)	T3 (139)	*P*-value^ϼ*^
		Mean ± SE	Mean ± SE	Mean ± SE	
Macronutrients					
Energy (Kcal/d)	2000–2200	2056 ± 103	2108 ± 106	2624 ± 106	< 0.001
Fat (g/d)	44–77	46.5 ± 1.3	56.6 ± 1.3	68.3 ± 1.3	< 0.001
Protein (g/d)	46–66	56.2 ± 0.9	67.1 ± 0.9	81.0 ± 0.9	< 0.0001
Carbohydrates (g/d)	130	359.8 ± 4.8	382.4 ± 4.9	374.1 ± 5.0	0.005
Methyl donor nutrients					
Vitamin B2 (mg/d)	1.2	0.4 ± 0.01	0.6 ± 0.01	0.9 ± 0.01	< 0.001
Vitamin B6 (mg/d)	1.4	1.0 ± 0.02	1.2 ± 0.02	1.4 ± 0.02	< 0.001
Vitamin B9 (mg/d)	400	545.8 ± 16.02	653.1 ± 16.44	729.9 ± 16.51	< 0.001
Vitamin B12 (µg/d)	2.4	0.7 ± 0.10	1.4 ± 0.10	2.7 ± 0.10	< 0.001
Methionine (mg/d)	14 mg/Kg	261.1 ± 27.16	505.5 ± 27.89	835.2 ± 28.00	< 0.001
Betaine (g/d)	9–15	75.5 ± 6.10	128.9 ± 6.27	184.8 ± 6.29	< 0.001
Choline (mg/d)	425	435.5 ± 11.47	502.8 ± 11.78	586.7 ± 11.83	< 0.001
Other nutrients					
Iron (mg/d)	15–18	11.3 ± 0.29	12.6 ± 0.30	13.5 ± 0.30	< 0.001
Ca (mg/d)	7000 − 1000	347.9 ± 9.60	460.9 ± 9.86	605.6 ± 9.90	< 0.001
Zinc (mg/d)	8	5.0 ± 0.09	5.6 ± 0.09	6.1 ± 0.09	< 0.001
Food groups					
Cereals (g/d)		615.2 ± 9.23	604.1 ± 9.48	586.5 ± 9.52	0.099
Grains (g/d)		110.2 ± 8.28	116.8 ± 8.50	136.3 ± 8.53	0.081
Vegetables (g/d)		103.4 ± 7.44	100.3 ± 7.63	86.6 ± 7.67	0.262
Green leafy vegetables (g/d)		22.2 ± 4.71	60.97 ± 4.83	102.3 ± 4.85	< 0.001
Dairy/milk products (g/d)		37.4 ± 4.84	67.1 ± 4.97	95.9 ± 4.99	< 0.001
Meat 9 g/d)		12.8 ± 3.98	33.7 ± 4.08	76.2 ± 4.10	< 0.001
Nuts (g/d)		1.9 ± 1.50	6.2 ± 1.54	4.0 ± 1.55	0.132
Fruits and Juices (g/d)		33.7 ± 5.92	50.9 ± 6.08	73.5 ± 6.11	< 0.001

*Mean values and standard error, n = 421. All values were adjusted for energy intake (Kcal/d)

^ϼ*^P-values were extracted from Residual Model Tests.

^9*^ RDA refers to the dietary recommended allowance

Participants in the third tertile of methyl donor nutrients had a lower prevalence of depression, anxiety, and stress compared to women in the first tertile (32.2% vs. 37.0%; 32.2% vs. 36.8%; and 31.5% vs. 38.5%, respectively) **(**Table 3**)**.

**Table 3:** The prevalence of depression, anxiety and stress across tertiles of methyl donor nutrients among study participants*

Tertiles of methyl donor micronutrients									
Variables	Whole population		T1(n = 145)		T2 (n = 137)		T3 (139)		*P*-value**
	N (%)		N (%)		N (%)		N (%)		
Depression	332 (78.9)		123 (37.0)		102 (30.7)		107 (32.2)		0.083
Anxiety	342 (81.2)		126 (36.8)		106 (31.0)		110 (32.2)		0.091
Stress	314 (74.6)		121 (38.5)		94 (29.9)		99 (31.5)		0.009

*values refer to numbers and percentages, n = 421

**P-values were obtained from Chi-Square tests.

Table 4 shows the multivariable-adjusted odds ratios for depression, anxiety and stress by tertile of methyl donor nutrients. We observed that top tertile of methyl donor nutrients do not have significant role against psychological disorders in both crude and adjusted models (depression, OR = 0.95, CI: 0.48; 1.88; anxiety, OR = 0.88, CI: 0.43, 1.79; stress, OR = 0.73, CI: 0.38, 1.40) (*p* > 0.05).

**Table 4:** Multivariable-adjusted* odds ratios for depression, anxiety and stress across tertiles of methyl donor nutrients

Tertiles of methyl donor micronutrients									
Variables	T1(n = 145)		T2 (n = 137)			T3 (139)			*P*-value**
	1		OR	CI		OR	CI		
**Depression** Crude Adjusted	1		0.52 0.72	0.28, 0.94 0.38, 1.37		0.59 0.95	0.32, 1.09 0.48, 1.88		0.103 0.975
**Anxiety** Crude Adjusted	1		0.51 0.72	0.27, 0.96 0.37, 1.42		0.57 0.88	0.30, 1.07 0.43, 1.79		0.092 0.804
**Stress** Crude Adjusted	1		0.43 0.57	0.24, 0.76 0.31, 1.07		0.49 0.73	0.27, 0.86 0.38, 1.40		0.720 0.427

* (Odds Ratios and 95% confidence interval) adjusted for energy intake (Kcal/day), marital status, education level, settlement area, financial level, and BMI

** *P*-values were obtained from binary logistic regression model.

While conducting the linear regression analyses we observed no association between methyl donor nutrients intake and psychological disorders (*p* > 0.05) **(**Table 5**)**.

**Table 5:** Linear associations of methyl donor nutrients intake in relation to depression, anxiety and stress

Variables	Per tertiles of methyl donor nutrients*							
	Beta		95% CI			*P*-value**		R-Square
Depression	-0.16		-0.35, 0.03			0.102		0.006
Anxiety	-0.17		-0.37, 0.02			0.009		0.006
Stress	-0.21		-0.39, -0.03			0.013		

*Tertiles of methyl donor nutrients were used as ordinal variables.

**P-values were obtained using linear regression models.

## Discussion

The current cross-sectional study showed that women within the highest tertile of methyl donor nutrient consumption had significantly higher intakes of protein, carbohydrate, fat, energy, B vitamins (B2, B6, B9, B12), methionine, betaine, choline, iron, zinc and calcium. Conversely, they had lower intakes of cereals and vegetables compared to women in the first and second tertiles.

Consistent with these findings, a cross-sectional study showed that people in the top quartile of methyl donor micronutrient scores (MDMS) had higher intakes of protein and fat [8]. However, they also had lower intakes of energy and carbohydrates [8], which is inconsistent with our results. Another cross-sectional study showed that people scoring in the highest tertile of B6 and B9 intake, had higher intakes of grains, proteins and carbohydrates, but lower fat intake. People in the top tertile of B12 had higher intakes of grains and protein, but lower intake of carbohydrates [25]. Inconsistent with our study, one cohort study found that people in the highest quantiles of B6, B9 and B12 had lower energy intakes [26].

Our study also showed that the prevalence of stress was higher among women in the highest tertile of methyl donor nutrients compared with women in the first two tertiles. However, we did not find significant inverse association between methyl donor intake and these mental disorders in both crude and adjusted models.

Several other studies are consistent with our findings [25–27]. A cross-sectional study of women showed that neither B6 nor B12 were significantly associated with anxiety. There was also no significant association between B9 and depression, anxiety or psychological distress [25]. Another cross-sectional study among women showed no significant associations between vitamins B6, B9 or B12 with prevalence of depression in adjusted models [26]. In addition, a cross-sectional study of Japanese workers found no significant association between folate intake and depressive symptoms in women [27].

In addition, a double-blind, randomized, placebo-controlled trial showed no treatment effect of B12 on depression [28]. A Finnish prospective study of middle-aged men did not observe any association between dietary B2, B6 and B12 intake and psychological disorders [29]. Furthermore, a cohort study showed no association between B2, B6, B9 and B12 intake and psychological distress in women aged 36 or 43 years [30]. In accordance with our results, a longitudinal study in older men and women indicated that dietary intakes of B6, B9 and B12 were not linked to depression [31]. A large population-based cross-sectional study of men and women aged 46–49 and 70–74 years in the Hordaland Homocysteine Study found that plasma choline concentration was not significantly associated with depressive symptoms [32].

Other research has shown results that are inconsistent with our findings. A cross-sectional study of Iranian adults showed that women in the highest quartile of the MDMS had lower odds of anxiety, depression and psychological distress after adjustment for covariates [8]. Another cross-sectional study also indicated a significant inverse association between vitamin B6 and B12 intakes and odds of depression in adjusted models among women. Similarly, it showed that in adjusted models B12 intake was positively associated with the odds of psychological distress [25]. A Finnish prospective study on middle-aged men indicated that there was a significant association between B9 intake and depressive symptoms [29]. Similarly, a cross-sectional study of Korean people aged 65 years and older found that lower folate and vitamin B12 levels may be risk factors for depression in later life [33]. Moreover, a cohort study of British women revealed that B12 intake was linked to psychological distress at age 53 [30]. Another study found some evidence of a reduced risk of depression associated with higher dietary intake of B6 in older women [34]. A meta-analysis also suggest that dietary intake of B2, B6, and B12 may be inversely associated with the risk of depression, an association which was significant in females [11]. inconsistent results between studies may be due to differences in study populations, dietary patterns, study designs, and inadequate control for confounding. Some studies were conducted in elderly people who tend to have poor absorption of some nutrients including B12, which may influence he results [28, 31, 33, 34]. In addition, mental disorders were also assessed in distinct ways, which may explain differences across studies.

The focus of the current study on reproductive-aged women was due to the fact that the prevalence of depression is higher among women according to national and international statistics [35, 36]. In addition, according to the WHO, studies on women are limited [36]. Moreover, women’s fertility might be affected by their mental health status [37]. To the best of our knowledge, this is the first study to examine the association of methyl donor nutrients and mental disorders including stress, anxiety and depression among reproductive-aged women. Previous studies have mostly focused on B group vitamins. In the current study, we extended this research to include methyl donor nutrients, which consist of two other nutrients (choline and betaine) in addition to B2, B6, B9 and B1. Limitations of this study include its cross-sectional nature and the fact that it was conducted only in Kabul city among women of reproductive age in government health centres using the convenience method. Therefore, the results of this study cannot be generalized to the entire population of Afghanistan. Despite having some limitations, the strengths of this study is the use of DASS 21, to measure mental health problems and it is a novel study that represents the association of methyl donor with mental health problems in reproductive age women of Afghanistan.

In the present study, despite the fact that no significant association was found in women, we conclude that methyl donor nutrients may play a protective role against common mental disorders. Further prospective studies and clinical trials are needed to confirm these findings.

## Data Availability

The datasets used and analyzed during the current study are available from the corresponding author on reasonable request.
